# A Benign Cardiac Growth but Not So Indolent

**DOI:** 10.1155/2016/8794202

**Published:** 2016-05-16

**Authors:** Adil S. Wani, Sahadev T. Reddy, Lakshmi Harinath, Robert W. W. Biederman

**Affiliations:** ^1^Department of Medicine, Allegheny General Hospital, Pittsburgh, PA 15212, USA; ^2^Gerald McGinnis Cardiovascular Institute, Allegheny General Hospital, Pittsburgh, PA 15212, USA; ^3^Department of Pathology, Allegheny General Hospital, Pittsburgh, PA 15212, USA

## Abstract

Cardiac lipomatous hypertrophy is a rare benign condition that usually involves the interatrial septum. Due to its benign nature it rarely requires intervention. Its presence outside the interatrial septum is reported infrequently. We present a case of lipomatous hypertrophy in the intraventricular septum that was complicated by a severe, symptomatic, and disabling dynamic left ventricular outflow tract obstruction. The symptoms significantly improved following the excision of the mass. In our case transthoracic echocardiogram was used to visualize the mass and measure the severity of the obstruction; Cardiac Magnetic Resonance Imaging was used to characterize the mass and histopathology confirmed the diagnosis.

## 1. Introduction

Cardiac lipomatous hypertrophy is a rare condition, which was first described in 1964 involving the interatrial septum (IAS). It is rarely diagnosed because of lack of specific clinical symptoms. The reported incidence on autopsy series was 1–2.2%. Histologically, cardiac lipomatous hypertrophy differs from a lipoma by its location in the IAS, absence of a capsule, and the presence of fetal fatty tissue. Its presence in the myocardium outside of the IAS has been reported very infrequently. Furthermore, it is typically a benign finding requiring no intervention. We present a case of lipomatous hypertrophy in the intraventricular septum complicated by severe, symptomatic, and disabling dynamic left ventricular outflow tract obstruction.

## 2. Case Description

A 54-year-old Caucasian male presented with eight-month history of progressively worsening shortness of breath. Five years before, on a routine physical examination, he was found to have a harsh systolic murmur along the left upper sternal border for which he underwent a transthoracic echocardiogram (TTE). The TTE showed mild systolic anterior motion of the mitral valve with significant left ventricular outflow tract gradient. A definite septal abnormality could not be excluded based on the TTE. Subsequently, he underwent a cardiovascular magnetic resonance imaging (CMR) to characterize the lesion and was found to have a basal anterior septal mass measuring 15.2 × 5.9 mm. The signal intensity by T1 and T2 was consistent with fatty tissue. Moderate LVOT acceleration was also noticed in the vicinity of the septal mass. The LVOT gradient was further evaluated by an exercise stress echocardiography which demonstrated a resting LVOT gradient of 10 mmHg that significantly increased to 69 mmHg at 79% of maximal predicted heart rate and returned to baseline at a heart rate of 88 bpm. It also showed a significant bileaflet systolic anterior motion of mitral valve at peak heart rate as compared to the baseline without any significant mitral regurgitation. No intervention was performed due to lack symptoms and good functional capacity. He was followed up with a CMR every other year, which showed small increments in size but he remained clinically asymptomatic.

**Figure 1:**
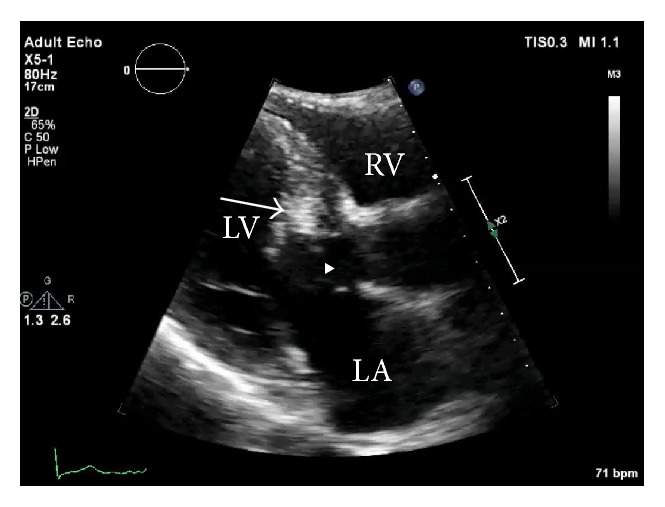
Preoperative Transthoracic Echocardiogram, Parasternal Long Axis. LA: left atrium; LV: left ventricle; RV: right ventricle; arrow: interventricular septal mass; arrow head: left ventricular outflow tract.

**Figure 2:**
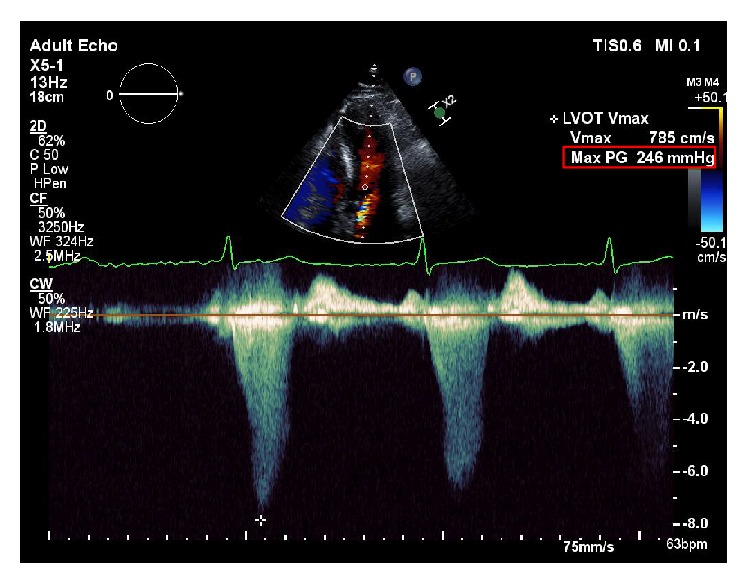
Preoperative Transthoracic Echocardiogram demonstrating left ventricular outflow tract pressure gradient of 246 mmHg with Valsalva.

**Figure 3:**
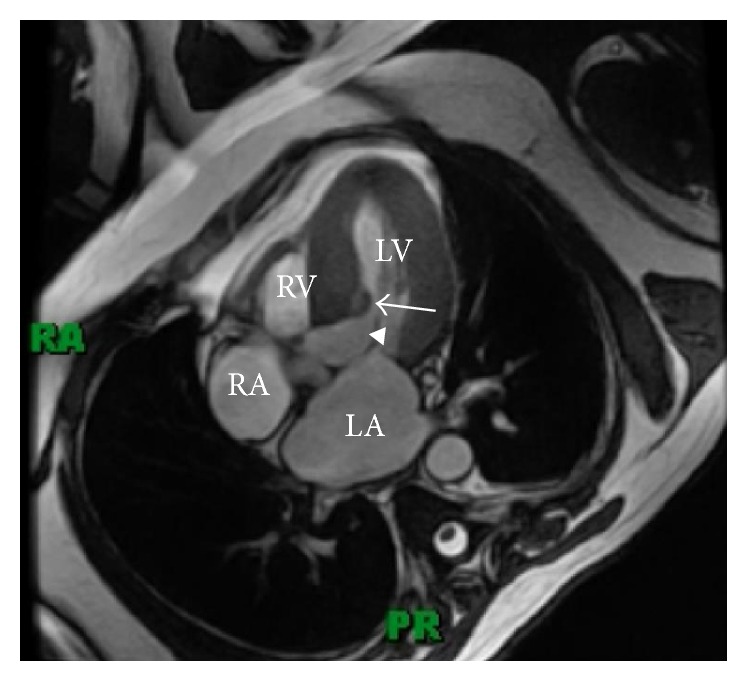
Preoperative Cardiac MR, 4-chamber view. LA: left atrium; LV: left ventricle; RA: right atrium, RV: right ventricle; arrow: interventricular septal mass; arrow head: left ventricular outflow tract.

Eight months prior to this report, he started experiencing dyspnea with exertion which progressively worsened. During this presentation his cardiac examination demonstrated a loud crescendo-decrescendo 5/6 systolic ejection murmur along left sternal border that worsened with Valsalva. A TTE was performed which demonstrated the previously visualized nonmobile septal mass (Figure 1) and a trace mitral regurgitation. It also demonstrated a marked increase in the peak systolic pressure gradient across the LVOT reaching 246 mmHg with Valsalva compared to the previous echo (Figure 2). His blood pressure on that day was 142/60 mmHg. Taking this into account his left ventricular peak systolic pressure was >350 mmHg. There must have been a component of contamination by the mitral regurgitation, but irrespective of that he undoubtedly had a significantly high pressure gradient across the LVOT. For better tissue characterization, a CMR was repeated which demonstrated an interval increase in the size of mass to 21 × 7 mm (Figure 3). Postgadolinium images did not show any evidence of uptake and there was no involvement of the subendocardium or the valves. It also showed a moderate mitral regurgitation with posteriorly directed jet along with LVOT turbulence.

The patient was referred for surgical evaluation and he underwent a surgical resection of the mass via transaortic approach. Intraoperatively the mass was identified as a fibrofatty tissue, which was resected without any complications. His postoperative course was uneventful. His symptoms significantly improved after the surgery permitted him to resume work.

Histopathological examination of the excised specimen showed benign hypertrophic myocardial muscle cells which revealed variation in the size and shapes of the nuclei. These myocytes were dispersed in between unencapsulated mature adipocytes with vacuolated cytoplasm. There were no mitotic figures or signet ring structures and the adipocyte nuclei were not hyperchromatic or indented, which ruled out liposarcoma (Figures 5(a): low power and 5(b): magnified).

**Figure 4:**
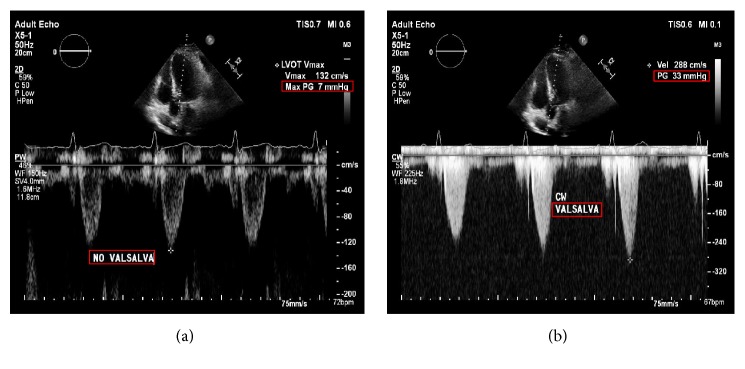
Postoperative Transthoracic Echocardiogram demonstrating resting left ventricular outflow tract pressure gradient of 7 mmHg (a) and a pressure gradient of 33 mmHg on Valsalva (b).

**Figure 5:**
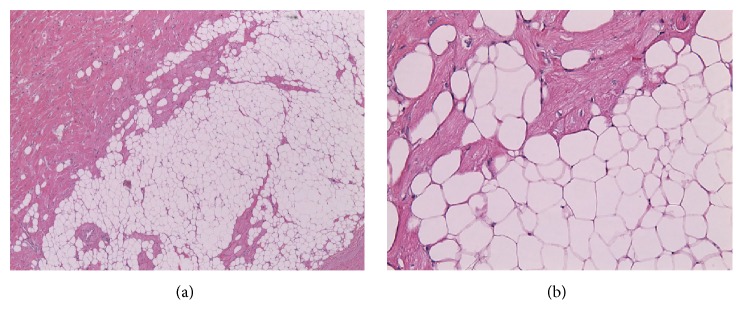
Adipose tissue interspersed with hypertrophic cardiac muscle, low power on the left (a) and high power on the right (b) demonstrating mature adipocytes with vacuolated cytoplasm.

At the two-month follow-up visit he felt exceptionally well and had resumed all his activities without any limitation. A repeat echocardiogram showed a resting pressure gradient of 7 mmHg (Figure 4(a)) and peaking at 33 mmHg with Valsalva maneuver (Figure 4(b)) that was significantly improved from the presurgical gradient of 246 mmHg.

## 3. Discussion

Fatty deposition of the heart is very common especially in the western world. Most common sites include the subepicardial region particularly adjacent to coronary arteries; right atrial and ventricular wall; atrial septum; areas of scarring in left ventricular free wall; infrequently the left atrial wall; and extremely rare in the interventricular septum [1–3]. Lipomatous hypertrophy (LH) is the exaggerated growth of the fatty tissue infiltrating into the myocardium. It can be differentiated from the lipoma by its lack of discrete capsule and the composition of both mature adipocytes and fetal fat cells. This abnormality is more common than a cardiac lipoma and is typically associated with obesity and aging [4].

Lipomatous hypertrophy of the interatrial septum has been described commonly in the literature [5]. It is mostly diagnosed on an autopsy and also as an incidental finding on TTE examination. Fyke III et al. described 17 cases, with the most common presenting symptom being palpitations. Several of the cases also presented with angina. Arrhythmias (mostly supraventricular) and even sudden cardiac death had been reported in a few cases.

Involvement of interventricular septum with LH is extremely rare. Verberkmoes et al. [4] described a 71-year-old woman with a membranous ventricular septal defect who was found to have a 40 × 35 mm echo-dense mass in the right atrium that intraoperatively was found to involve the atrial septum, left atrium, and the right ventricle. Resected tissue on pathological examination showed lipomatous hypertrophy. Nezafati et al. [6] reported a 27-year-old woman with a systolic murmur who was found to have a nonencapsulated echogenic mass in the interventricular septum extending in the right ventricular outflow tract. Histologic study revealed “benign lipomatous hypertrophy.” Heyer et al. [7] described a large hyperechogenic mass protruding into the right ventricular cavity and the outflow tract, which was homogenous and had an infiltrating pattern on the Cardiac MR. Pathologic examination showed adipocyte proliferating between the hypertrophic cardiac myocytes without any fibrous capsule covering the mass.

Tissue characterization of the cardiac masses is difficult to establish on an echocardiogram. Cardiac CT and MR due to their high spatial resolution have been shown to be superior in characterizing intracardiac masses [7, 8]. A fat attenuation on a cardiac MRI can help to exclude myxoma, rhabdomyoma, fibroma, and/or mesothelioma. A homogenous mass with high signal density similar to subcutaneous fat has been found to be suggestive of a LH [9].

Although dynamic left ventricular outflow tract obstruction is a common association with hypertrophic cardiomyopathy (HOCM), it has never been reported to be caused by a lipomatous hypertrophy. An LVOT blood outflow acceleration due to the presence of an obstruction causing lift and drag resulting in the systolic anterior motion of the mitral valve (Venturi Effect) can explain the high peak aortic pressure gradient as seen in our case [10]. Surgical management is infrequently performed in patients with minimal symptoms. Surgery should be limited to LH complicated by severe rhythm disorders, superior vena-cava syndrome, outflow obstruction, or altered hemodynamic cardiac function leading to congestive heart failure [11]. The combination of echocardiography and CMR to define the anatomy and physiology of this rare case of “malignant” lipomatous hypertrophy were instrumental and underscore their use in rare cases of seemingly benign cardiac masses.
